# An integrated analysis and comparison of serum, saliva and sebum for COVID-19 metabolomics

**DOI:** 10.1038/s41598-022-16123-4

**Published:** 2022-07-13

**Authors:** Matt Spick, Holly-May Lewis, Cecile F. Frampas, Katie Longman, Catia Costa, Alexander Stewart, Deborah Dunn-Walters, Danni Greener, George Evetts, Michael J. Wilde, Eleanor Sinclair, Perdita E. Barran, Debra J. Skene, Melanie J. Bailey

**Affiliations:** 1 Faculty of Engineering and Physical Sciences, University of Surrey, Guildford, GU2 7XH UK; 2 Faculty of Health and Medical Sciences, University of Surrey, Guildford, GU2 7XH UK; 3 Surrey Ion Beam Centre, University of Surrey, Guildford, GU2 7XH UK; 4 Frimley Park Hospital, Frimley Health NHS Trust, Frimley, GU16 7UJ UK; 5 School of Geography, Earth and Environmental Sciences, University of Plymouth, Plymouth, PL4 8AA UK; 6 Manchester Institute of Biotechnology, University of Manchester, Manchester, M1 7DN UK

**Keywords:** Lipidomics, Metabolomics, Biomarkers, Diseases, Infectious diseases

## Abstract

The majority of metabolomics studies to date have utilised blood serum or plasma, biofluids that do not necessarily address the full range of patient pathologies. Here, correlations between serum metabolites, salivary metabolites and sebum lipids are studied for the first time. 83 COVID-19 positive and negative hospitalised participants provided blood serum alongside saliva and sebum samples for analysis by liquid chromatography mass spectrometry. Widespread alterations to serum-sebum lipid relationships were observed in COVID-19 positive participants versus negative controls. There was also a marked correlation between sebum lipids and the immunostimulatory hormone dehydroepiandrosterone sulphate in the COVID-19 positive cohort. The biofluids analysed herein were also compared in terms of their ability to differentiate COVID-19 positive participants from controls; serum performed best by multivariate analysis (sensitivity and specificity of 0.97), with the dominant changes in triglyceride and bile acid levels, concordant with other studies identifying dyslipidemia as a hallmark of COVID-19 infection. Sebum performed well (sensitivity 0.92; specificity 0.84), with saliva performing worst (sensitivity 0.78; specificity 0.83). These findings show that alterations to skin lipid profiles coincide with dyslipidaemia in serum. The work also signposts the potential for integrated biofluid analyses to provide insight into the whole-body atlas of pathophysiological conditions.

## Introduction

Since 2020, the COVID-19 pandemic has been at the forefront of global health. Great strides have been made in fields as diverse as diagnosis, treatment and vaccination^1,2^. Nonetheless, SARS-CoV-2 still poses a major health risk to the world. This reflects the difficulty of eradicating all disease reservoirs, as well as the potential for the virus to evolve new variants of concern^3^, potentially leading to vaccine escape^4^. Furthermore, as the illness becomes endemic^5^, better understanding of COVID-19 pathologies and appropriate treatments will continue to be a focus for the healthcare community. Whilst the major symptoms of COVID-19 are now well-described, the metabolic processes underlying these changes are incompletely understood. This is not least because of the unusually wide range of these symptoms, which have also changed as new variants have emerged^6^.

Metabolomics offers insight into the drivers of different pathologies, but reflecting the urgency of conducting research in a pandemic, most metabolomics research to date has focused on biomarker discovery rather than on validation^7,8^. Furthermore, the vast majority of biomarker discovery research for COVID-19 and other diseases has been carried out using blood as a sampling matrix. As a biofluid rich in metabolites not prone to external contamination, blood sampling offers considerable advantages in terms of biological interpretation, offering diagnostic power via clinical indicators such as lymphocytes as well as metabolites^9,10^. No single biofluid, however, can provide insight to all potential pathologies. For example, patients with COVID-19 have been reported to suffer a variety of cutaneous manifestations, including chilblains, maculopapular lesions, urticarial lesions, vesicular lesions and other rashes and manifestations^11,12^. A large-scale study carried out in the UK found a significant association between skin rashes and a positive swab test result; 17% of COVID-19 positive cases reported skin rashes as the first clinical sign of COVID-19, and 21% reported rashes as the only clinical sign of COVID-19^13^. The disease has also been reported to cause oral manifestations, separate to those related to respiratory disorders, such as dry mouth, mucosal lesions and oral fungal infection^14,15^. Whilst these cutaneous and oral symptoms have been widely reported, their metabolic causes are unknown. Furthermore, whilst blood-based metabolomic changes have been well-described, investigation of COVID-19 induced changes in the skin lipidome and the salivary metabolome have to date been few in number and restricted to untargeted mass spectrometry methods or infra-red spectroscopy studies that do not provide full identification of biomarkers^16–18^.

**Figure 1:**
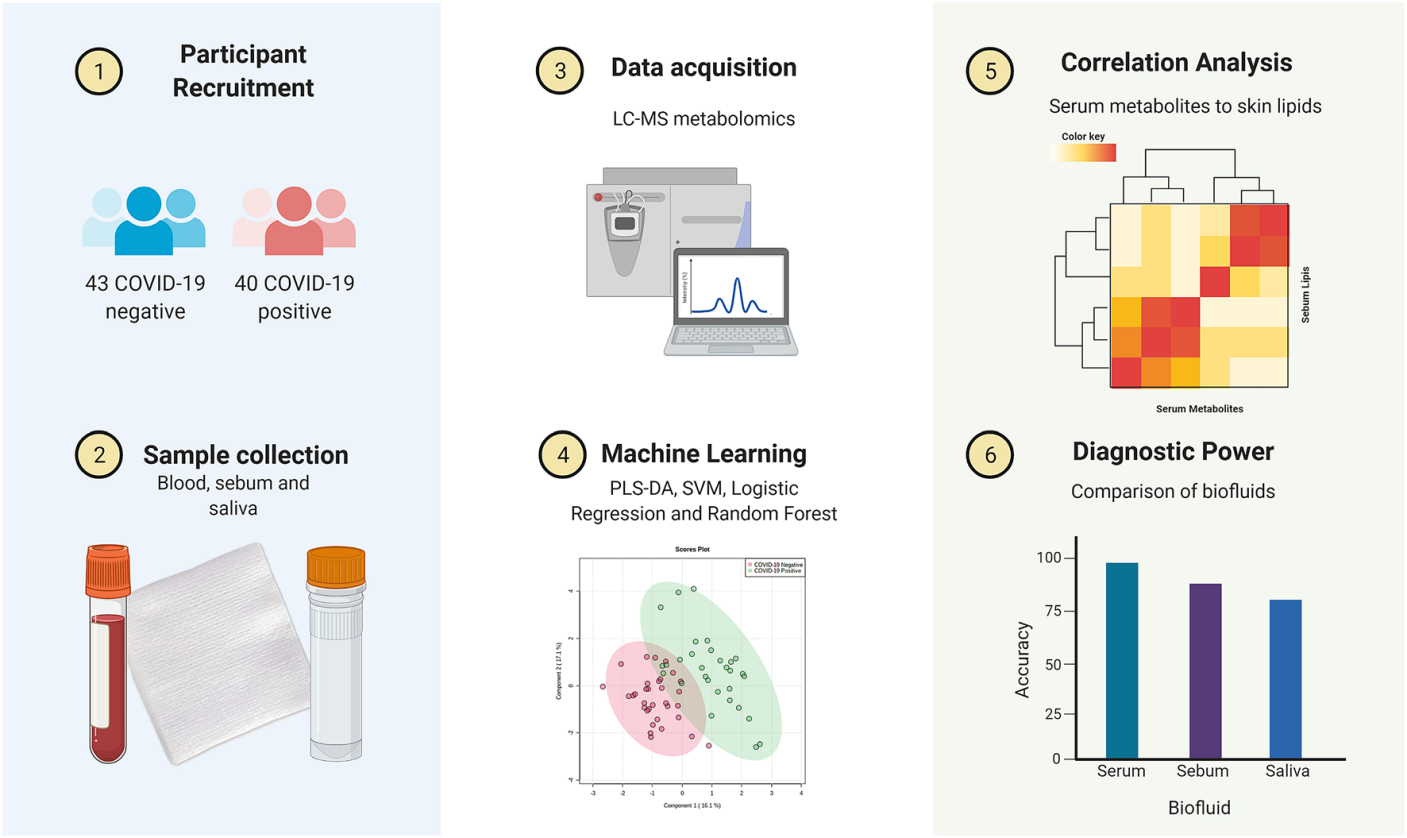
Integrated metabolomics for discovery and validation of COVID-19 biomarkers, created with BioRender.com.

In this study, correlation analysis was performed between serum, sebum and saliva features to provide a more holistic view of COVID-19 related dysregulation (a summary of the workflow is presented in Fig. 1). Integrated analyses incorporating different biofluids have greater ability to capture biological complexity and have helped the understanding of COVID and other illnesses^19,20^. A recent study by Pozzi et al. provided an integrated analysis of salivary and serum metabolites^21^, finding that a combined ‘omics approach could differentiate between COVID-19 inpatients and outpatients. To our knowledge, however, no integrated analysis of sebum, saliva and serum for COVID-19 or indeed other illnesses has yet been performed. This study aims to fill this gap. This is both to investigate potential linkages between biofluids given evidence of skin-related and oral symptoms, and also to investigate more generally the relationships between biofluids and how those relationships might be perturbed.

In the context of metabolomics using different biofluids as sampling matrices, it should be noted that for diagnosis of COVID-19, blood-based analyses have outperformed less-invasive analysis such as breath, sebum or saliva. One meta-analysis found that blood-based analyses delivered average sensitivity of 0.89 and specificity of 0.96, compared with 0.76 and 0.81 across the less invasive biofluids^8^. Meta-analysis of independent studies does not, however, allow for comparison of diagnostic power to be tested on an identical participant population. In addition to correlation analysis, therefore, in this work the relative capabilities of serum, sebum and saliva in differentiating COVID-19 positive cases from controls was also compared. This was done to allow for direct comparisons of potential diagnostic power, a comparison which to our knowledge has not previously been performed.

## Materials and methods

### Participant recruitment and ethics

Ethical approval for this project (IRAS project ID 155921) was obtained via the NHS Health Research Authority (REC reference: 14/LO/1221). The participants included in this study were recruited consecutively (i.e. without selective sampling criteria other than suspicion of COVID-19 infection) at Frimley Park NHS Trust, UK. The study recruited 83 participants between May 2020 and July 2020. Participants were identified by clinical staff to ensure that they had the capacity to consent to the study and were asked to sign an Informed Consent Form based on the International Severe Acute Respiratory and emerging Infection Consortium/World Health Organisation (ISARIC/WHO) Clinical Characterisation Protocol for Severe Emerging Infections. Those patients that did not have this capacity were not sampled. Signatures were witnessed by University of Surrey researchers. At the time of recruitment, participants were categorised by the hospital as either “query COVID” (meaning there was clinical suspicion of COVID-19 infection, but a negative positive RT-PCR SARS-CoV-2 test result had been recorded during their admission) or “COVID positive” (meaning that a positive test result had been recorded). All participants were provided with a Patient Information Sheet explaining the goals of the study. All methods performed as part of this study were performed in accordance with the relevant guidelines and regulations.

### Sample collection, extraction and processing

Collection of the samples was performed by researchers from the University of Surrey at Frimley Park NHS Foundation Trust hospitals; collection took place on admission or in some cases shortly afterwards. Participants were requested to provide all three biofluids, but due to declined consent for blood sampling, or inability to express saliva or easily provide blood, not all participants provided all three biofluids (Table S1). All samples (sebum, saliva and serum) were taken from each patient within 20 min of one another. Alongside biofluid collection, metadata for all participants was also collected covering *inter alia* sex, age, comorbidities (based on whether the participant was receiving treatment), the results and dates of COVID-19 PCR (polymerase chain reaction) tests, bilateral chest X-Ray changes, smoking status, and whether the participant presented with clinical symptoms of COVID-19. Values for lymphocytes, CRP and eosinophils were also taken—here values within five days of biofluid sampling were recorded.

Serum collection and extraction followed the protocols set out by the COVID-19 Coalition^22^. In brief, venous blood was collected in 3 mL serum tubes, transported to University of Surrey by courier whilst stored on ice, and centrifuged on arrival at 1600×*g* for 10 min at 4 °C. All samples with a sampling time interval greater than four hours were rejected. Serum was then decanted into 100 µl aliquots and stored at − 80 °C until processing. Prior to analysis, the serum was sterilised using 200 µl of ethanol into 100 µl of serum (2:1 v/v solvent/sample ratio). Saliva collection and extraction was performed as described in Frampas et al.^16^, again following the protocols established by the COVID-19 MS Coalition^22^. Finally, sebum collection and extraction was performed as described in Spick et al.^17^, using an untargeted liquid-chromatography mass spectrometry (LC–MS) methodology.

Serum samples were analysed using the Biocrates MxP Quant 500 system using a Xevo TQ-S Triple Quadrupole Mass Spectrometer coupled to an Acquity UPLC system (Waters Corporation, Milford, MA, USA). The MxP Quant 500 system provides targeted quantification of metabolites including amino acids and derivatives, bile acids, biogenic amines, acylcarnitines, carbohydrates and other small molecule metabolites, plus a wide array of lipids. Analysis takes place via a single assay, and two analytical procedures. The first of these procedures operates by liquid chromatography (operated in both positive and negative ion mode) and the second by flow injection analysis (positive ion mode), both coupled to tandem mass spectrometry with isotopically labelled internal standards for quantification. Sample order was randomised across 96-well plates, and 3 levels of quality controls (QC) were run on each plate. Blank PBS (phosphate-buffered saline) samples (three technical replicates) were used for the calculation of the limits of detection (LOD). Biogenic amines and amino acids were quantified for each plate using a seven-point calibration curve, with other analytes semi-quantitated with a single point standard (i.e. assuming concentration linearity in the range measured). The levels of metabolites present in each QC were compared to the expected values and the CV% calculated. Data were normalised between the three batches using the results of quality control level 2 (QC2) repeats across the plate (n = 5) and between plates (n = 3) using Biocrates METIDQ software (QC2 correction). Metabolites where > 25% concentrations were at or below the limit of detection (≪ LOD), above the limit of quantification (> LOQ), or where the blank was out of range were excluded (total n excluded in serum = 150). The remaining 474 quantified metabolites comprised of 8 acylcarnitines, 20 amino acids, 26 biogenic amines, 11 bile acids, 53 ceramides, 15 cholesteryl esters, 1 cresol, 9 diglycerides, 4 carboxylic and fatty acids, 85 phosphatidyl cholines, 14 sphingolipids, 222 triglycerides, 2 hormones, 2 indoles, 1 nucleobase and 1 vitamin.

Saliva samples were quantified in a similar way to the serum samples but employing the Biocrates AbsoluteIDQ p180 system, which can detect up to 188 metabolites, fewer than the MxP Quant 500. The method used was as described for the MxP Quant 500 system, i.e. the sample order was randomised, the same 3 levels of QCs were run on each 96-well plate, and the same protocols were followed to establish CVs, LODs and LOQs. As for serum, metabolites where > 25% concentrations were at or below LLOQ, where measurements were above LOQ, or where the blank was out of range were excluded (total n excluded in saliva = 103). The remaining 83 quantified metabolites comprised 7 acylcarnitines, 18 amino acids, 5 biogenic amines, 42 glycerophospholipids and 11 sphingolipids.

Sebum samples were analysed as set out described in Spick et al.^17^, using an untargeted liquid-chromatography mass spectrometry (LC–MS) methodology using a Dionex Ultimate 3000 HPLC module operated in reverse phase mode with a C18 column, coupled to an Orbitrap Q-Exactive Plus mass spectrometer operating in positive ion mode. Features with a coefficient of variation (CV%) across all pooled QCs above 20% were removed, as were those that were not present in at least 90% of pooled QC injections. These features were then field blank adjusted: all features with a signal to noise ratio below 3 × were also rejected, leaving 998 features deemed to be robust and reproducible for analysis.

### Feature identification

In this study identifications were made in accordance with the Metabolomics Standards Initiative for metabolite identification^23^. Serum and salivary metabolites were identified and quantified using isotopically labelled internal standards, retention times and multiple reaction monitoring. The mass spectrometry conditions were used as optimised and provided by Biocrates. Sebum features in this work were either identified as putatively annotated compounds based on accurate *m/z* matching, or were unknown compounds with differentiable and quantifiable MS signals but no library identification. For all three biofluids, the output was a data block in the form of a peak:area matrix of *n* participants by *p* features.

### Statistical analysis

Initial pre-processing of the three data blocks was conducted in Progenesis QI (for sebum samples) and using manufacturer software (for the Biocrates kits) as described in the preceding section. This pre-processing generated three data blocks in the comma separated values (.csv) format. Missing value replacement for the data blocks was carried out using the K-nearest neighbours algorithm^24^. All data were then log transformed, mean centred and pareto scaled using the web-based platform MetaboAnalyst^25^, specifically the Statistical Analysis (One Factor) module of the platform. Analysis of the processed peak:area matrices was then conducted in MetaboAnalyst together with user-written scripts in the programming language Python and the package scikit-learn^26,27^. Participant characteristics (positive and negative cohorts) were assessed by two-tailed t-tests, or by two-tailed Mann Whitney *U* tests where parameters were not normally distributed.

To explore relationships between the different sampling matrices, correlation analysis by Pearson correlation coefficient was conducted across the data blocks to measure the strength of the relationship between the features in the three biofluids, where the correlation coefficient *r* ranges from + 1 (perfect positive correlation) to − 1 (perfect negative correlation). This analysis was performed for all possible combinations of putatively identified sebum features and identified salivary and serum metabolites.

To compare the ability of the different sampling matrices to differentiate between positive and negative COVID-19 participants, logistic regression using recursive feature elimination with cross validation (RFECV) was performed in Python using the scikit-learn package for each block, inclusive of metabolic ratios previously identified as diagnostic (serum and saliva ratios of kynurenine/arginine, kynurenine/tryptophan and glutamine/glutamate). RFECV was performed to limit the feature set employed and reduce overfitting^28^, leading to a set of smaller data blocks to be analysed. RFECV is a feature selection algorithm which takes the entire feature set, constructs a model, and then removes selected features to test whether predictive accuracy of the model improves or deteriorates. The algorithm repeats this process of removing features iteratively until it reaches a local maximum for cross-validated prediction accuracy.

Partial least squares-discriminant analysis (PLS-DA) was then conducted for each of the three data blocks (serum, sebum and saliva) using the reduced feature set. PLS-DA is a supervised multivariate technique that reduces high-dimensional data into a smaller number of orthogonal components which can be used to represent the full dataset^29^. In the case of metabolomics, many thousands of features can be reduced into a small number of components, which can then be used to classify and make predictions about the status of participants. Leave-one-out cross-validation (LOOCV) was used for model validation to test accuracy, sensitivity and specificity using COVID-19 RT-PCR results as the ground truth. Sensitivity was defined as the true positive rate, i.e. the probability that a positive test result will be obtained when the disease is present, and calculated from a confusion matrix as true positives/(true positives plus false negatives). Specificity was defined as the true negative rate, i.e. the probability that a negative test result will be obtained when the disease is not present, and calculated as true negatives/(true negatives plus false positives).

Variable importance in projection (VIP) scores were used to assess feature/metabolite significance. The VIP feature scores are based on the amount of variance between the positive and negative participants that is explained by each feature across the components. To all intents and purposes, VIP scores reflect the relative importance of a feature in classifying participants as cases or controls.

To identify potential confounders in the analysis, Principal Component Analysis (PCA)—an unsupervised multivariate technique—was used^30^. This approach has some similarities to PLS-DA but—as an unsupervised method—constructs a set of orthogonal components that represent the full dataset using the features showing the maximum overall variation, rather than variation between the conditions (controls versus COVID-19 in this case).

## Results

### Population metadata overview

The study population analysed in this work included 83 participants, comprising 40 participants presenting with a positive COVID-19 RT-PCR test and 43 participants with a negative RT-PCR test but presenting with similar clinical symptoms to COVID-19. A summary of the metadata is shown in Table 1. The populations providing sebum, saliva and serum samples each represented a subset of this group, as not all participants consented to provide all biofluids, or were unable to generate sufficient saliva or blood due to age or infirmity. Complete data by subgroup is shown in Supplementary Material, Table S1.

**Table 1:** Characteristics of study population.

Parameters	Negative for COVID-19	Positive for COVID-19	p-value
N	43	40	
Age (mean, standard deviation; years)	62.9 ± 19.2	61.4 ± 19.8	0.74
Male/female (n)	20/23	20/20	0.54
Treated for hypertension (n)	18	13	0.39
Treated for high cholesterol (n)	10	6	0.36
Treated for type 2 diabetes mellitus (n)	12	11	0.97
Treated for ischaemic heart disease (n)	7	5	0.64
Current smoker (n)	2	2	0.94
Ex-smoker (n)	13	6	0.11
Medical acute dependency admission (n)	7	13	0.09
Intensive care unit admission (n)	1	5	0.10
Survived admission (n)	41	37	0.62
Duration of pre-admission symptoms (mean, standard deviation; days)	11.9 ± 20.2	6.7 ± 6.8	0.12
Time between positive RT-PCR test and sampling (mean, standard deviation; days)	NA	5 ± 7	
Lymphocytes (mean, standard deviation; cells/μL)	1.0 ± 0.5	0.7 ± 0.3	0.002
C-Reactive Protein (mean, standard deviation; mg/L)	138.4 ± 96.4	170.8 ± 121.2	0.20
Eosinophils (mean, standard deviation; 100/μL)	0.3 ± 0.4	0.2 ± 0.3	0.007
Bilateral chest X-ray changes (n)	6	22	0.001
Continuous positive airway pressure (n)	4	10	0.07
O2 required (n)	14	21	0.07

Age and sex distributions for COVID-19 positive and negative cohorts were similar (mean age of 61.4 years and 62.9 years, and M:F ratios of 1.00 × and 0.87 × respectively). On average, participants had seen 8 days of pre-admission symptoms. Comorbidities are associated with both hospitalisation and more severe outcomes for COVID-19 infection, but will also alter the metabolome of participants, representing both a causative and confounding factor. Due to hospital recruitment, however, comorbidities including T2DM, hypertension, high cholesterol and ischaemic heart disease were represented in both the positive and negative groups. Ex-smokers and current smokers were more highly represented in the COVID-19 negative group (35% of the negative participants, versus 20% of the positive participants). All participants had at least a clinical suspicion of COVID-19 infection, thus respiratory distress due to past or present smoking may have caused ‘over-recruitment’ of smokers in the cohort that subsequently tested negative.

Levels of C-Reactive Protein (CRP) were higher for COVID-19 positive participants, whilst lymphocyte and eosinophils levels were lower. A two-tailed Mann Whitney U test on CRP levels, lymphocytes and eosinophils provided p-values of 0.20, 0.002 and 0.007, respectively. Effect sizes (calculated by Cohen’s D) were 0.28, − 0.39 and − 0.34, respectively. COVID-19 positive participants were also more likely to present with bilateral chest X-ray changes, more likely to require oxygen/CPAP, and were also escalated to ICU and MADU more frequently. These observations were in agreement with literature descriptions of COVID-19 symptoms, clinical indicators and progression^31^.

### Feature identification

For serum and saliva, metabolite identification was performed under manufacturer protocol using internal standards together with accurate mass matching, also allowing for quantification of concentrations. For saliva the AbsoluteIDQ p180 system generated 83 identified metabolites that were reliably quantified (out of a theoretical maximum of 188). For serum the MxP Quant 500 system generated 472 identified metabolites that were reliably quantified in samples (out of a theoretical maximum of 630). For sebum, an untargeted lipidomics approach was used, with accurate mass matching using the Progenesis QI software, with tandem MS where possible. A total of 998 sebum features (both putatively identified lipids and unidentified compounds) were considered to be reliable and robust, as set out in Spick et al.^17^. A limited metabolite set was also investigated for serum to provide an equivalent comparison of diagnostic accuracy for serum versus saliva (i.e. to remove the advantage of the serum peak:area matrix being more feature-rich than saliva).

### Correlation analysis: serum and sebum lipids

**Figure 2:**
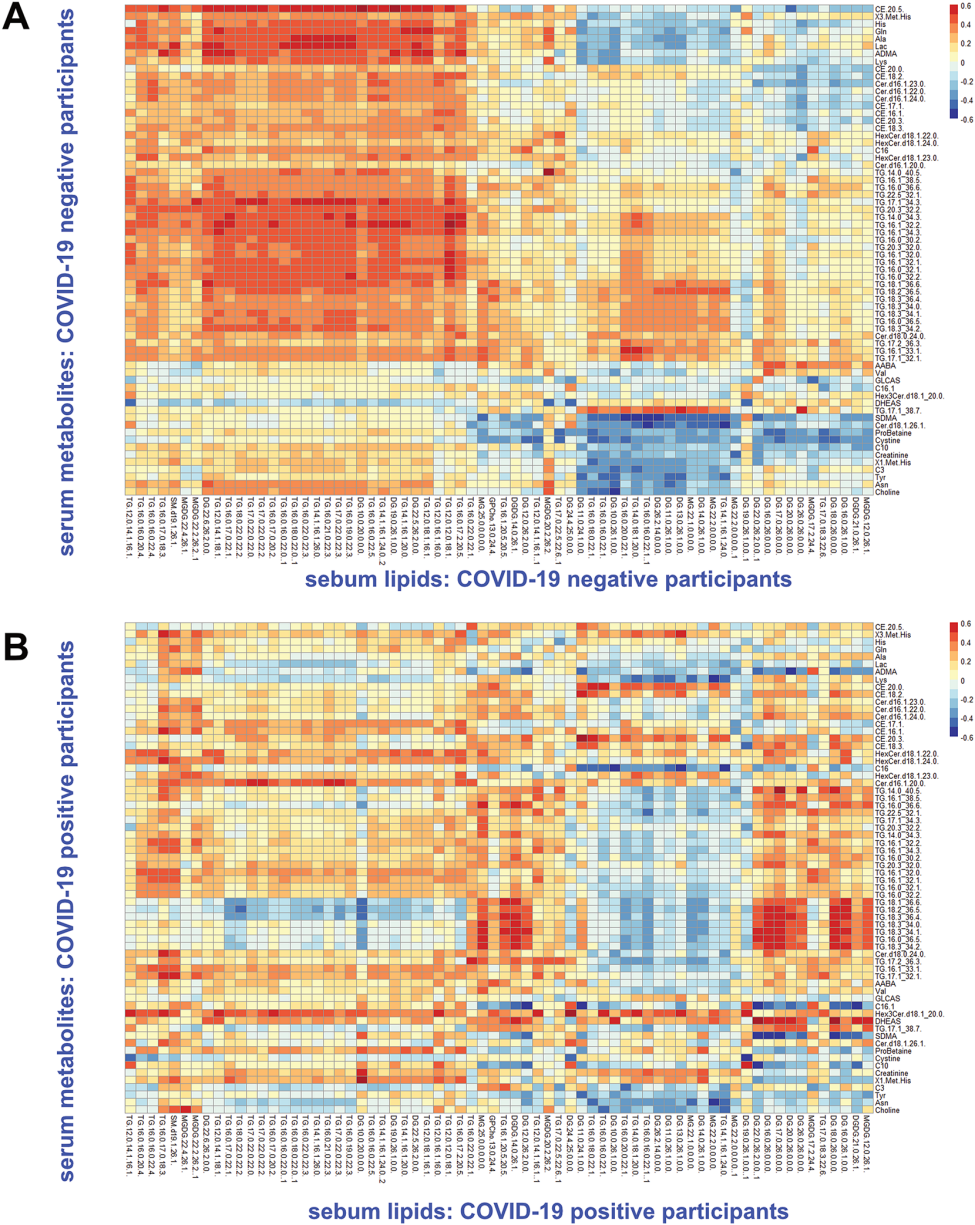
Heatmaps showing Pearson correlation coefficients between sebum lipids and serum metabolites sampled from study participants: COVID-19 negative participants (**A**) and COVID-19 positive participants (**B**).

Whilst sebum was analysed via an untargeted LC/MS method, and so the putative features identified cannot be matched against direct serum metabolites, correlations between the sebum and serum data blocks were investigated. This was done by correlating every possible combination of sebum and serum features in each of the matrices. For each pairing of sebum features and serum metabolites, the Pearson correlation coefficient *r* was calculated. The average absolute value of *r* for all pairings of named features in sebum and serum was 0.11. No significant difference in this average absolute value of *r* was observed between COVID-19 positive participants and controls, but the patterns of lipid correlation were significantly altered. Figure 2 shows heatmaps of the calculated Pearson correlation coefficients for a subset of metabolites and lipids with the strongest overall relationships between the sebum and serum data blocks, split between COVID-19 negative (Fig. 2A) and positive (Fig. 2B) participants.

**Figure 3:**
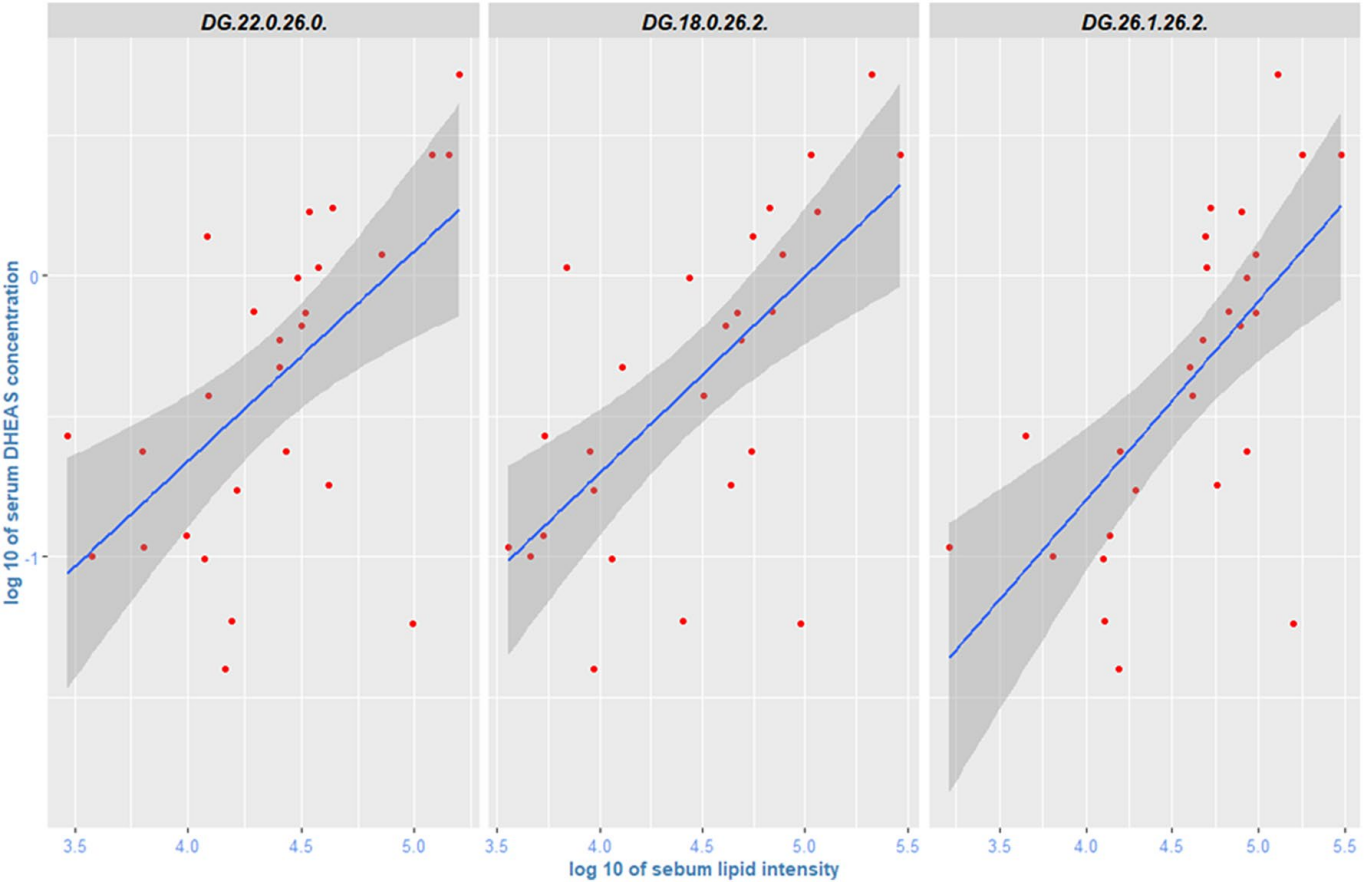
Pairwise correlations between serum DHEAS and sebum diglycerides (COVID-19 Positive study participants).

Correlation coefficients in Fig. 2 show clustering, and some of the observed correlations are relatively high, reaching 0.6. As can be seen in the lower part of Fig. 2A for COVID-19 negative participants, several serum carnitines (especially hexadecenoylcarnitine) and l-Proline Betaine correlate negatively with sebum lipids. There is also a set of general and relatively strong positive correlation coefficients (0.5 to 0.6) between serum triglycerides and several sebum glycerides, visible on the left side of Fig. 2A. In the cohort of COVID-19 positive participants, however, this general pattern breaks down (Fig. 2B), and different serum metabolites show increased/decreased correlation with sebum glycerides. Figure 3 illustrates the positive correlation between serum DHEAS (dehydroepiandrosterone sulphate) and three specific diglycerides in sebum for COVID-19 positive participants; these correlations were markedly weaker in COVID-19 negative participants.

### Correlation analysis: serum and saliva

**Figure 4:**
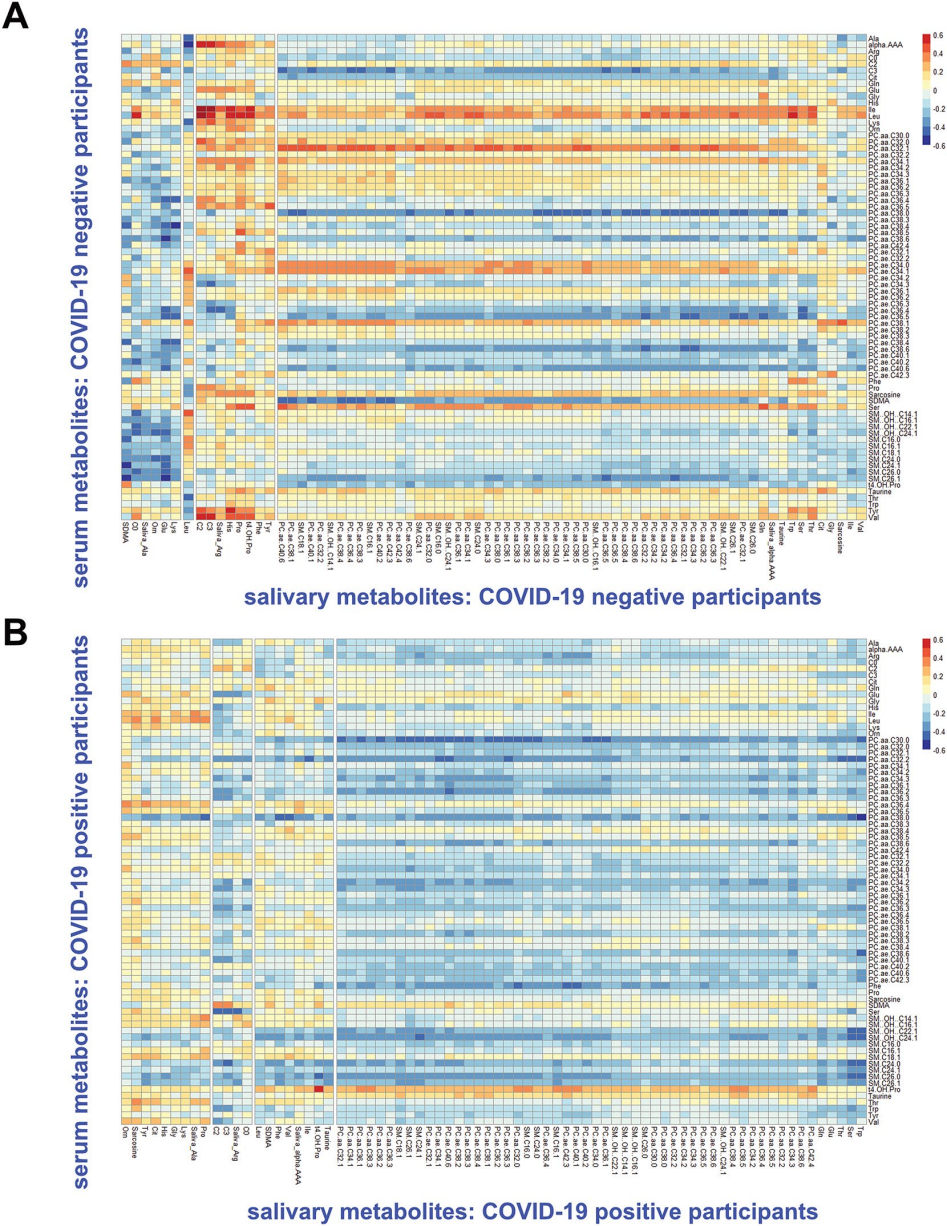
Heatmaps showing Pearson correlation coefficients between serum and salivary metabolites: sampled from study participants: COVID-19 negative participants (**A**) and COVID-19 positive participants (**B**).

Both serum and saliva samples were processed using a standardised Biocrates platform employing internal standards for accurate quantification of concentrations. This allowed investigation of the extent of correlation of salivary and serum concentrations for 79 identified metabolites for the same patients, both directly (correlation of specific metabolites in serum versus saliva) and generally (overall correlation between the two data blocks). The average Pearson correlation coefficient *r* for paired metabolites (e.g. taurine in serum versus taurine in saliva) was negligible for both COVID-19 positive and negative participants; only one paired correlation coefficient was outside the range of 0.2 to − 0.2, this exception being trans-4-hydroxyproline (t4-OH-Pro). Some serum metabolites showed a general pattern of positive correlation in the COVID-19 controls, such as serum leucine and isoleucine being generally associated with higher concentrations of salivary metabolites (Fig. 4A). These positive correlations were reduced in COVID-19 positive participants (Fig. 4B), and correlations between serum and saliva were weaker than those observed for serum and sebum.

### Diagnostic performance

To investigate the dysregulation due to COVID-19 in different biofluids, the ability of each of the biofluids to differentiate between positive and negative cases was analysed by PLS-DA analysis of their respective reduced feature data blocks, and sensitivity/specificity/accuracy for each data set was calculated together with confidence intervals. Two models were constructed for serum—one with the full Biocrates MxP Quant 500 metabolite set, and one using a metabolite set equivalent to the p180 Biocrates system to allow for a more comparable assessment versus the saliva PLS-DA model.

As can be seen from Table 2, the best performance was delivered by serum using a reduced panel of 41 metabolites, with sensitivity of 0.97 (95% confidence interval of 0.83–1.00) and specificity of 0.97 (0.84–1.00). Restricting the serum feature set to metabolites detected by the p180 Biocrates kit lowered sensitivity to 0.83 (0.65–0.94) and specificity to 0.94 (0.80–0.99). Sebum saw a major reduction in features using RFECV, from 998 to 26, and the resulting PLS-DA model achieved sensitivity of 0.92 (95% confidence interval of 0.78–0.98) and specificity of 0.84 (0.69–0.93). Saliva delivered a PLS-DA model with sensitivity of 0.78 (95% confidence interval of 0.56–0.93) and specificity of 0.83 (0.63–0.95), performing less well than the other biofluids. The reduced data sets were also investigated for confounders by PCA; neither age nor sex showed clustering for serum, saliva or sebum (Figs. S2–S4, Supplementary Material).

**Table 2:** Comparison of PLS-DA model performance across three different biofluids using leave-one-out-cross-validation to assess performance.

Biofluid	*n* patients (pos/neg)	*p* features /after RFECV^b^	Sensitivity	Specificity	Accuracy ^a^	R2Y	Q2Y
Saliva	47 (23/24)	83/5	0.78	0.83	0.80	0.42	0.26
Sebum	80 (37/43)	998/26	0.92	0.84	0.88	0.63	0.51
Serum	63 (30/33)	472/41	0.97	0.97	0.97	0.90	0.72
Serum (p180 only)	63 (30/33)	86/23	0.83	0.94	0.89	0.78	0.47

^a^All PLS-DA models used 5 components except saliva which found maximum accuracy with 3 components.

^b^Number of features in model is the smaller number after recursive feature elimination with cross-validation.

**Figure 5:**
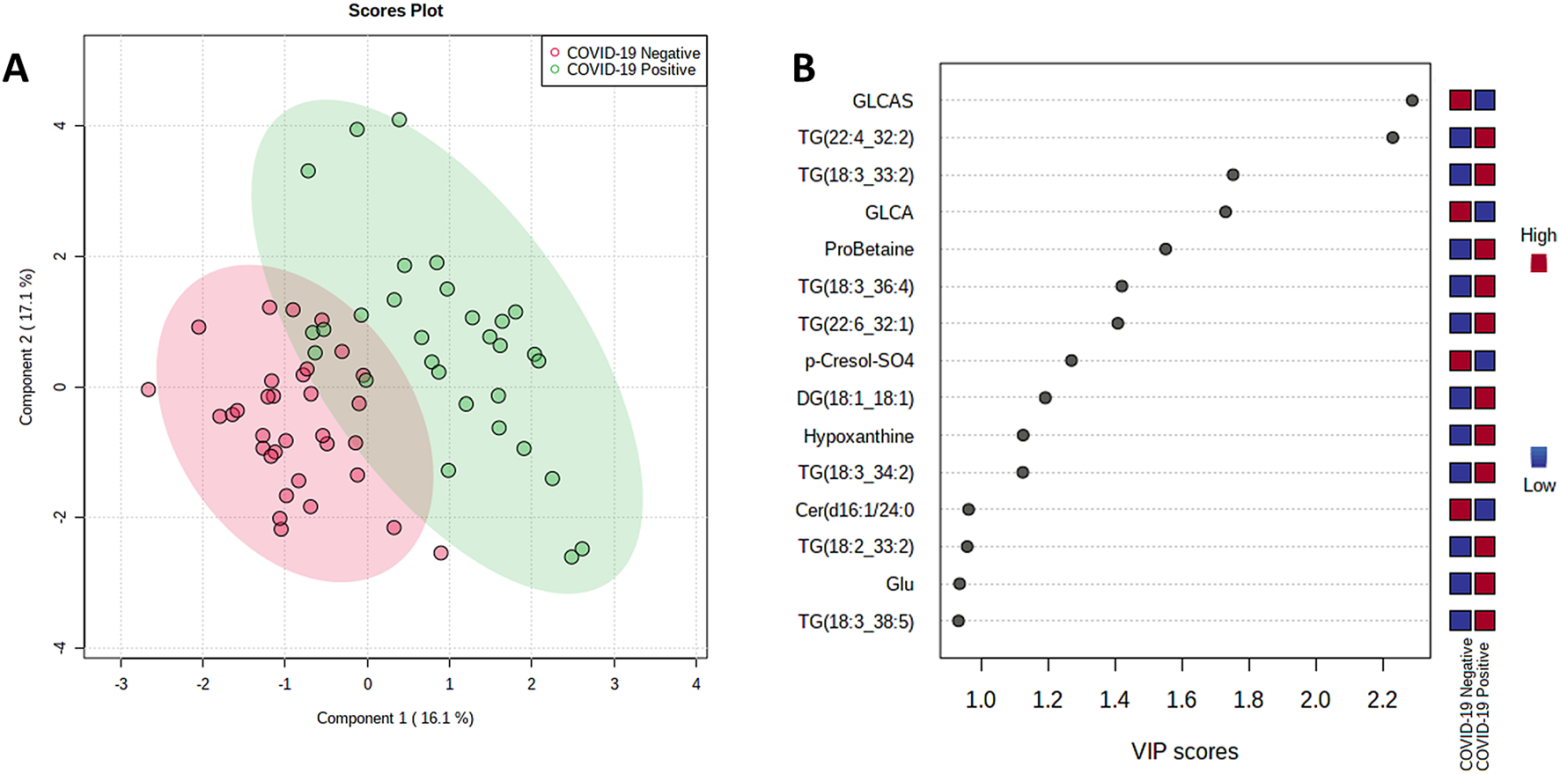
(**A**) PLS-DA plot and (**B**) high VIP score metabolites for serum, COVID-19 positive versus COVID-19 negative.

The datasets were additionally processed by support vector machine (SVM), logistic regression and random forest models, and in all cases, comparable sensitivity and specificity was achieved (Table S2, Supplementary Material), confirming that separation is not sensitive to the model used. Figure 5 shows separation of COVID-19 positive and negatives for serum by PLS-DA as well as the highest VIP score metabolites.

**Figure 6:**
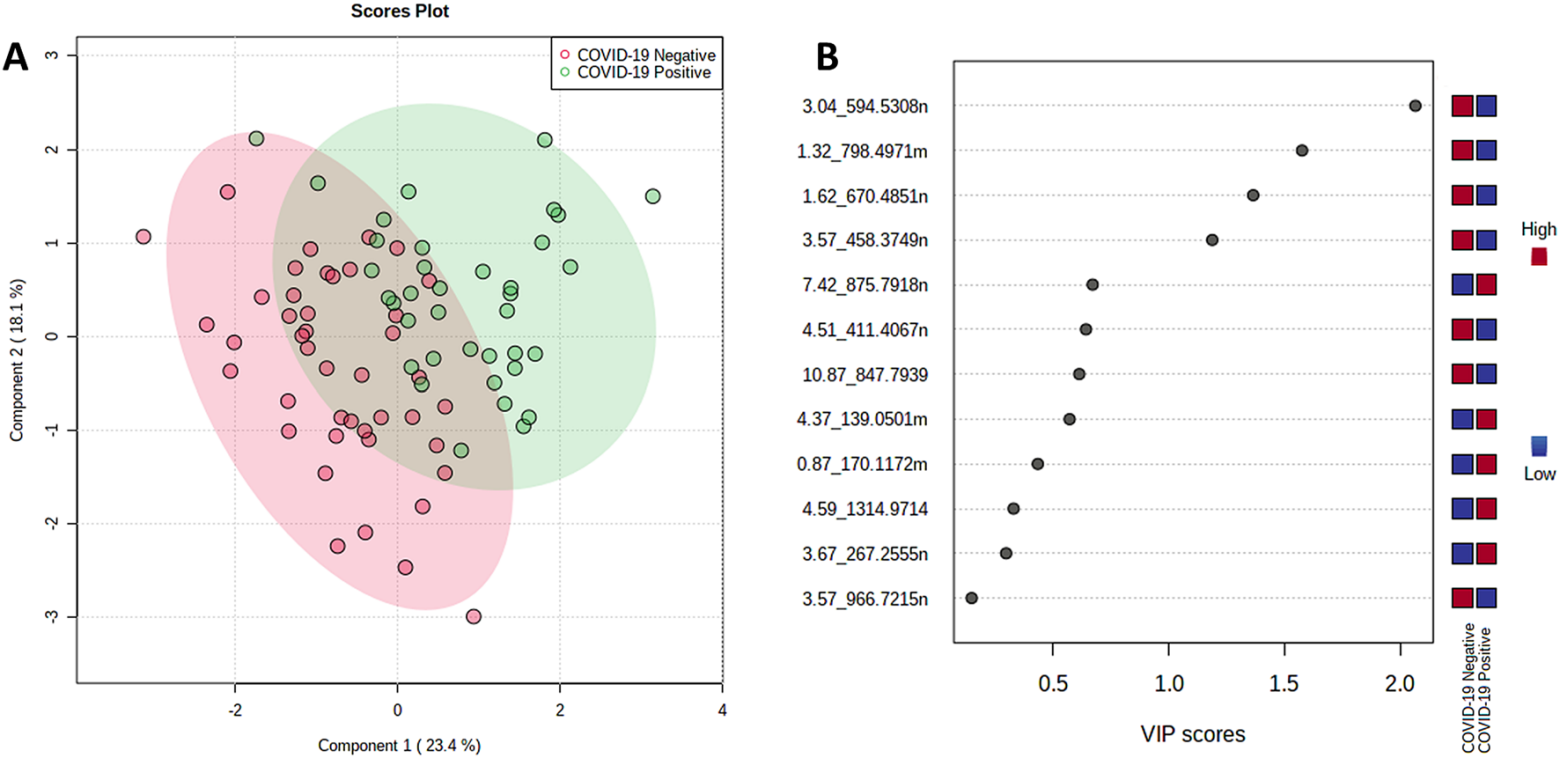
(**A**) PLS-DA plot and (**B**) high VIP score metabolites for sebum, COVID-19 positive versus COVID-19 negative.

**Figure 7:**
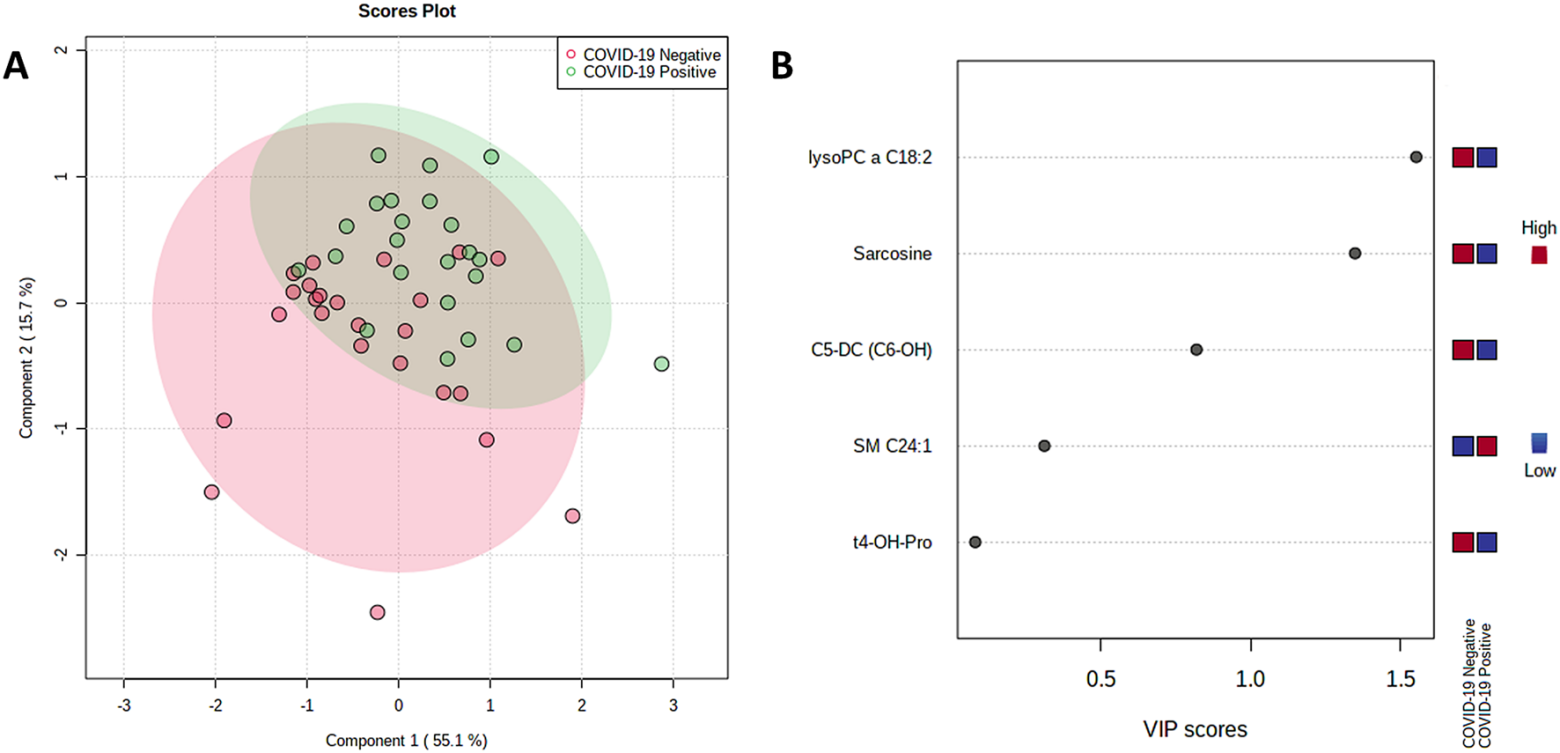
(**A**) PLS-DA plot and (**B**) high VIP score metabolites for saliva, COVID-19 positive versus COVID-19 negative.

Separation for serum can be seen visually in Fig. 5A with two components, rising to 0.97 accuracy with 5 components. The high VIP score metabolites are dominated by lipids, especially triglycerides, but the highest VIP metabolite was glycolithocholic acid 3-sulfate (GLCAS), a conjugated bile acid. Of the amino acids, L-proline betaine had the highest VIP score (Fig. 5B). Separation by PLS-DA is also shown below for both sebum (Fig. 6) and saliva (Fig. 7). Separation becomes visually worse in the order serum/sebum/saliva, with saliva also featuring a notably small number of high VIP metabolites, reflecting the difficulty of RFECV finding a local maximum for accuracy with a noisy dataset.

Finally, whilst for correlation analysis and for assessing important metabolites/classification, the largest possible populations have been used, it is also important to compare classification accuracy for the exactly matched 37 participants for which all three samples were taken (Fig. S1, Supplementary Material). For this subset of 37 participants, classification accuracy for serum, sebum and saliva (measured by LOOCV) was 0.95, 0.87 and 0.70, the same ranking as shown in Table 2 and almost identical classification accuracy.

## Discussion

To date, the vast majority of multi-omics studies of COVID-19 have focused on single biofluids, typically via proteomic and metabolomic analyses of blood^32^. Whilst blood-based pathway alterations are now well-described, especially dyslipidemia, amino acid dysregulation^33^, inflammatory responses, neutrophil activation and degranulation, and platelet degranulation^34^, other biofluids have not been investigated to the same degree. In this study sebum lipids showed patterns of strong correlation with serum metabolites in controls, but with major changes in COVID-19 positive participants, revealing disease-driven dysregulation in both biofluids. Notably, in the control group of COVID-19 negative participants, a set of positive correlations between serum triglycerides and ceramides and skin lipids was visible. This positive correlation in the controls was dysregulated in the cohort of COVID-19 positive participants, evidence that dyslipidemia due to COVID-19 is widespread. The integrated analysis presented here also showed correlation between sebum lipids and DHEAS in the cohort of COVID-19 positive participants. DHEAS is an immune-system positive adrenal hormone, an antiglucocorticoid and also a sex hormone. Alterations in any DHEAS / sebum axis could be indicative of immune response, and may underpin diagnostic differences seen in the sebum of COVID-19 positive and negative participants^35,36^. Pathways incorporating DHEAS and other sex hormones are also believed to play a role more widely in in human inflammatory skin diseases. Whilst the results presented in this work cannot show the direction of causality, in cutaneous manifestations of COVID-19 the relationship between sebum dysregulation and DHEAS may offer treatment opportunities^11,37^. Sebum lipids have been identified as biomarkers in other pathologies, such as Parkinson’s Disease where sebum dysregulation has been linked to carnitines^38,39^, but sebum as a biofluid of interest is less well researched than blood-based metabolomics. The results here suggest that sebum lipidomics holds promise for investigating other pathophysiological conditions.

Saliva showed weaker correlation to serum, especially in the case of directly matched metabolites. COVID-19 positivity did change the correlation maps between the two biofluids, but from a less correlated starting point than sebum / serum, and resulting in weaker diagnostic power overall. As a filtrate, saliva should be influenced by serum levels, but concentrations are lower than in blood^40^. Furthermore, the salivary biome is independent and has its own discrete functions and is markedly more subject to direct contamination from food or medication. Indeed, the correlation of metabolites between saliva and blood has previously been found to be weak or in some cases non-existent^41,42^.

The analysis of the relative ability of each biofluid to differentiate between COVID-19 positive participants and controls showed declining accuracy for the biofluids in the order serum (diagnostic accuracy 0.97), sebum (0.88), and saliva (0.80). Serum therefore performed best in this comparison of matched biofluids, but sebum also performed relatively well, and in this reanalysis better than previously reported^17^. This was in large part due to the use of feature reduction: the original sebum dataset’s 998 features are likely to have led to overfitting (exceeding the number of samples by a multiple of 15), worse generalisation and worse performance on cross-validation.

Accuracy was also investigated for serum using a more limited set of metabolites, equivalent to that provided by the p180 Biocrates kit, leading to reduced accuracy but still relatively better than the other biofluids. This finding illustrates the trade-off between narrowly targeted analyses and widely targeted (or untargeted) analyses—whilst it is easier to validate a more tightly controlled panel of metabolites, a wider range can reveal additional biomarkers, especially during the initial discovery phase of biomarker identification. The biomarkers responsible for separation between positive and negative measured by VIP score were: glycolithocholic acid 3-sulfate (GLCAS), a bile acid, two triglycerides (TG(22:4_32:2) and TG(18:3_33:2)), as well as the amino acid l-proline betaine. This is consistent with other studies finding evidence of dyslipidemia, particularly increased triglyceride levels^43–45^. The dysregulation of GLCAS is also concordant with liver damage caused by COVID-19^46^, and dysregulation of bile acids (deoxycholic acid and ursodeoxycholic/hyodeoxycholic acid) has previously been reported as a key feature specific to COVID-19, differentiating between COVID-19 and other respiratory and inflammatory diseases in hospital-recruited patients^47^.

Saliva performed least well in differentiating between COVID-19 positive from controls in this analysis. It should be noted, however, that due to the inability for ethical reasons to require abstinence from food or drink in the hospital setting, saliva would have been the most subject to environmental confounders such as the recent oral intake of food or medication. These factors may have confounded the study and limited insight into oral symptoms described in the literature. Whilst a clear limitation of the study, it does also reflect the practicalities of sampling during a pandemic or indeed in any busy clinic.

It should also be stressed that *n* for all three biofluids in this work was small, and so the comparisons of potential diagnostic power, i.e. ability to differentiate COVID-19 positive cases from controls, are indicative of relative performance only. Without a validation cohort the accuracies presented here should not be taken as indicative of absolute performance. A further limitation of the study resulting from small *n* is inability to match precisely by *inter alia* age and medication regime, all factors that affect metabolism. In addition, new variants have resulted in altered symptoms (this study was conducted when the Wildtype variant was dominant in the UK); vaccination status may also alter the impact of the disease on metabolism. It is to be hoped that larger studies, made possible through cohesive efforts such as the work of the MS Coalition, will be able to investigate these relationships and confounders more comprehensively in the future.

In conclusion, whilst the integrated analysis herein of serum, sebum and saliva shows challenges in identifying reproducible metabolic biomarkers of COVID-19, especially using saliva as a sampling matrix, it also shows the potential for non-invasive sampling in revealing relationships across biofluids and pathways. The correlation analysis presented in this study illustrates how serum metabolomics may provide insight into sebum dysregulation via a DHEAS/immune response mechanism, potentially offering insights into new treatments for that subset of COVID-19 patients suffering from cutaneous manifestations. For diagnostic purposes, however, where sensitivity and specificity are paramount, this work provides further evidence that blood-based metabolomics will remain the best-in-class approach.

## Supplementary Information

Supplementary Information.

## Data Availability

All data relating to this work will be made available on the Zenodo data repository following publication. The analytical protocols used as well as mass spectrometry .raw files, sample and participant data will be openly available for all researchers to access.
