# Supplementary material for: An integrated analysis and comparison of serum, saliva and sebum for COVID-19 metabolomics
Source: Sci Rep. 2022 Jul 13;12:11867. doi: 10.1038/s41598-022-16123-4 (PMC9278322; doi:10.1038/s41598-022-16123-4)
Supplement: Supplementary file 1 — Supplementary Information. [file 41598_2022_16123_MOESM1_ESM.docx]

**An integrated analysis and comparison of serum, saliva and sebum
for COVID-19 metabolomics: Supplementary Material**

**Table S1: Breakdown of patient population by biofluid**

| **Parameters** | **Serum** | | **Sebum** | | **Saliva** | |
| --- | --- | --- | --- | --- | --- | --- |
|  | **Negative** | **Positive** | **Negative** | **Positive** | **Negative** | **Positive** |
| N (positive / negative) | 33 | 30 | 37 | 43 | 24 | 23 |
| Age (mean, standard deviation; years) | 63.5 ±19.4 | 62.4 ±19.9 | 62.9 ± 19.2 | 61.3 ± 20.1 | 63.2 ± 22.4 | 53.5 ± 19.4 |
| Male / Female (n) | 17 / 16 | 17 / 13 | 20 / 17 | 19 / 24 | 11 / 13 | 16 / 7 |
| Treated for Hypertension (n) | 13 | 10 | 18 | 11 | 9 | 6 |
| Treated for High Cholesterol (n) | 7 | 5 | 10 | 5 | 7 | 4 |
| Treated for Type 2 Diabetes Mellitus (n) | 11 | 10 | 12 | 8 | 7 | 7 |
| Treated for Ischaemic Heart Disease (n) | 7 | 5 | 7 | 4 | 4 | 4 |
| Current Smoker (n) | 1 | 2 | 2 | 2 | 1 | 2 |
| Ex-Smoker (n) | 11 | 4 | 13 | 5 | 7 | 2 |
| Medical Acute Dependency admission (n) | 6 | 12 | 7 | 11 | 4 | 10 |
| Intensive Care Unit admission (n) | 1 | 4 | 1 | 5 | 1 | 3 |
| Survived Admission (n) | 32 | 28 | 35 | 40 | 23 | 22 |
| Duration of pre-admission symptoms (mean, standard deviation; days) | 8.0 ± 11.1 | 7.4 ± 7.3 | 11.9 ± 20.2 | 7.0 ± 6.9 | 7.9 ± 17.3 | 7.6 ± 8.0 |
| Gap between first RT-PCR and study sampling (mean, standard deviation; days) |  | 5.7 ± 6.3 |  | 5.2 ±6.1 |  | 5.2 ± 4.9 |
| Lymphocytes (mean, standard deviation; cells / μL) | 0.9 ± 0.5 | 0.6 ± 0.3 | 1.0 ± 0.5 | 0.7 ± 0.3 | 0.9 ± 0.5 | 0.7 ± 0.4 |
| C-Reactive Protein (mean, standard deviation; mg / L) | 129.8 ±  96.7 | 177.6 ± 123.3 | 138.4 ± 109.9 | 180.9 ± 118.9 | 133.7 ± 91.7 | 178.9 ± 123.2 |
| Eosinophils (mean, standard deviation; 100 / μL) | 0.4 ± 0.4 | 0.2 ± 0.4 | 0.3 ± 0.4 | 0.2 ± 0.3 | 0.3 ± 0.4 | 0.3 ± 0.4 |
| Bilateral Chest X-Ray changes (n) | 4 | 17 | 6 | 22 | 4 | 12 |
| Continuous Positive Airway Pressure (n) | 4 | 9 | 4 | 10 | 3 | 7 |
| O2 required (n) | 11 | 17 | 14 | 20 | 9 | 11 |

**
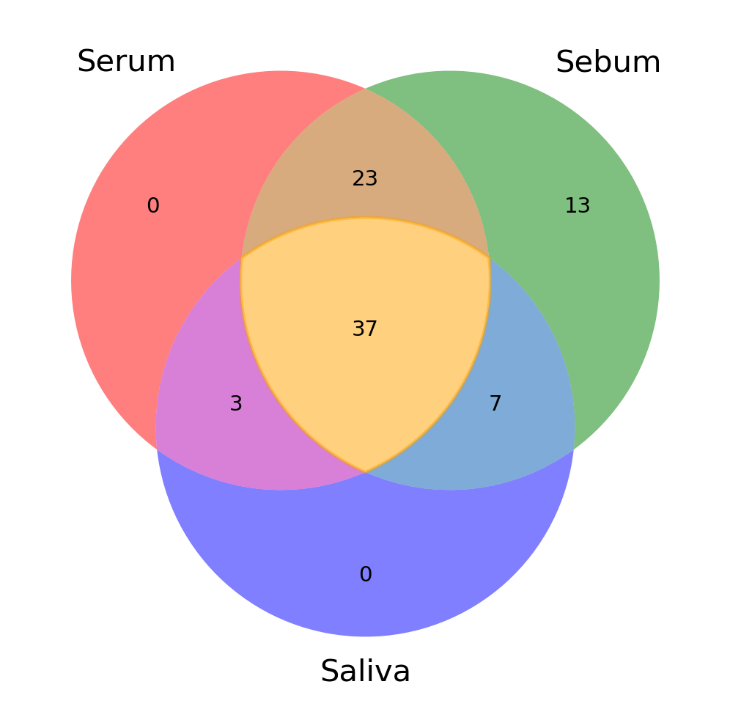
**

**Figure S1: Venn diagram illustrating samples obtained by biofluid**

**Table S2: Classification performance by machine learning algorithm**

| **Method** | **Serum Sensitivity** | **Serum Specificity** | **Sebum Sensitivity** | **Sebum Specificity** | **Saliva Sensitivity** | **Saliva Specificity** |
| --- | --- | --- | --- | --- | --- | --- |
| PLS-DA ^a^ | 0.97 | 0.97 | 0.92 | 0.84 | 0.78 | 0.83 |
| Random Forest ^b^ | 0.77 | 0.82 | 0.65 | 0.77 | 0.52 | 0.58 |
| Support Vector Machine ^c^ | 1.00 | 0.97 | 0.87 | 0.93 | 0.74 | 0.79 |
| Logistic Regression | 0.97 | 0.97 | 0.91 | 0.95 | 0.74 | 0.75 |
| Average of models | 0.93 | 0.93 | 0.84 | 0.87 | 0.70 | 0.74 |

^a^ As set out in Table 2

^b^ Number of trees = 500

^c^ Linear kernel, C = 10, gamma = 0.1

**Figure S2: Unsupervised analysis by PCA for serum – (A) age: 50 and over, Below 50 (B) sex: (Female, Male)**

**Figure S3: Unsupervised analysis by PCA for sebum – (A) age: 50 and over, Below 50 (B) sex: (Female, Male)**

**Figure S4: Unsupervised analysis by PCA for saliva – (A) age: 50 and over, Below 50 (B) sex: (Female, Male)**
